# Systematic prospective electrophysiological studies of the median nerve after simple distal radius fracture

**DOI:** 10.1371/journal.pone.0231502

**Published:** 2020-04-13

**Authors:** Pierre R. Bourque, John Brooks, Theo Mobach, Brendan Gammon, Steven Papp, Jodi Warman-Chardon

**Affiliations:** 1 Department of Medicine (Neurology), University of Ottawa, Ottawa, Canada; 2 Ottawa Hospital Research Institute, Ottawa, Canada; 3 Division of Orthopedic Surgery, University of Ottawa, Ottawa, Canada; Providence Care Hospital, CANADA

## Abstract

**Purpose:**

To assess whether there is a measurable impairment of median nerve conduction study parameters with uncomplicated distal radius fracture.

**Methods:**

Patients were assessed prospectively at the time of cast removal (visit 1) after a standard 6–8 week immobilization for uncomplicated distal radius fracture. Patients with prior entrapment neuropathy or polyneuropathy were excluded. Patients were asked to report sensory symptoms. Median and ulnar motor and sensory conduction studies were performed bilaterally, as well as transcarpal stimulation. All electrophysiologic studies were repeated at a follow-up visit 2, on average 7.8 weeks later.

**Results:**

39 patients were assessed at visit 1 and 30 (77%) were available for follow-up visit 2. Paresthesia in the median territory on the fractured side were reported in 20% at visit 1 and 26% at visit 2. Electrophysiological evidence of only mild carpal tunnel syndrome was found on the fractured side in 4/39 at visit 1 and 6/30 at visit 2. There were only 2 cases of moderate-marked median neuropathy, both asymptomatic and on the unfractured side. Median motor and sensory latencies and amplitudes did not show statistically significant differences between fractured and unfractured sides with the single exception of median distal motor latency at visit 1.

**Conclusions:**

Median territory paresthesia at the time of cast removal following distal radius fracture are often not associated with electrophysiologic evidence of median neuropathy. Most median nerve electrophysiologic parameters do not significantly differ between the fractured and uninjured sides. Significant traumatic median neuropathy is not likely to be a frequent manifestation of uncomplicated distal radius fracture.

**Level of evidence:**

Diagnostic analysis, Level III

## Introduction

Systematic prospective data is lacking to help determine to what extent a distal wrist fracture may affect median nerve function, as measured by commonly used electrophysiological techniques. Transient symptoms of carpal tunnel syndrome (CTS), spontaneously resolving within days or weeks, has been reported to occur in 4% of patients with distal radius fracture[1,2]. This must be contrasted with a more severe and disabling acute carpal tunnel syndrome (CTS), noted within hours, often after high energy trauma, with a reported frequency of 5.4% to 8.6%. CTS is more prevalent in patients requiring open reduction and internal fixation, particularly if there is an initial fracture translation greater than 35% [3]. Delayed CTS refers to a classical chronic median entrapment neuropathy appearing months to years after fracture, with a wide variation in reported prevalence ranging from 0.5 to 22%.

The literature has however mostly documented such time-dependent carpal tunnel syndromes based on the clinical assessment of symptoms and signs, which may have poor sensitivity and specificity[4]. Additionally, most reports did not compare their results to the incidental occurrence of median neuropathy on the non-fractured side. We report here the results of systematic and bilateral electrodiagnostic studies of the median and ulnar nerves in a cohort of 39 consecutive patients with distal radius fractures managed non-operatively.

The goal of our study was to determine whether a measurable impairment of median nerve function should be expected in the course of routine management of simple distal radius fracture.

## Methods

This study was conducted at The Ottawa Hospital during the 2016–2019 period and approved by Research Ethics Board (protocol 20160718-01H). It was part of a larger study to assess the effect of limb immobilization and disuse atrophy on hand intrinsic muscle electrodiagnostic parameters. All participants were over the age of 18 and provided written informed consent.

Patients were recruited at the time of cast removal for their distal radius fracture. We only included uncomplicated fractures not requiring surgical intervention. The usual care plan for such patients was a 6–8 week period of immobilization in a plaster or fiberglass cast applied to the forearm and wrist region. Patients were assessed for the study at the time of cast removal and a follow-up control visit 6–8 weeks after cast removal. The following exclusion criteria were applied: documented acquired polyneuropathy (e.g. diabetic or toxic/nutritional); hereditary polyneuropathy; prior surgery or significant trauma to the upper limb; history or treatment of entrapment of the median or ulnar nerve at the elbow or wrist.

The following data was collected: age, sex, handedness, side of bony fracture, duration of casting, interval between visits. At the time of cast removal (Visit 1) and 6–8 week follow-up (Visit 2) the presence and distribution of paresthesia in the fractured and non-fractured hand was noted. At both visits, the following electrophysiologic studies were performed: bilateral median (APB) and ulnar (ADM and FDI) motor nerve conduction studies, and bilateral median (index) and ulnar (little finger) sensory nerve conduction studies. To grade the severity median neuropathy, we followed the general outline of the AANEM guidelines [5,6], using the normative data determined at our own institution. Thus the nerve conduction study criteria for “mild” median neuropathy included normal motor and sensory amplitudes and the presence of either a median/ulnar digit 4 distal sensory latency difference greater than 0.5 ms or a transcarpal latency difference greater than 0.4 ms. A “moderate” median neuropathy at the wrist required prolonged sensory latencies and a distal motor latency greater than 4.5 ms. The definition of “severe” median neuropathy at the wrist required a median CMAP amplitude less than 4.5 mv (or a 50% relative reduction side-to-side) in addition to marked abnormalities in sensory conduction studies (low amplitude responses with very prolonged latency, or unobtainable responses).

### Electrophysiological techniques

All electrophysiological studies were performed with the Natus Nicolet EDX EMG (Natus Medical Incorporated, San Carlos CA). Different certified technologists could perform the assessments at different time points, but using identical protocols, techniques and equipment. The filter settings were 2 Hz-10 kHz for the motor nerve conduction studies (NCS) and 20 Hz-2 kHz for the sensory NCS. Stimulation was with a hand-held bipolar electrode, with an interelectrode distance of 3 cm and stimulus duration set at 0.1 millisecond. Skin temperature was maintained above 32°C bilaterally.

Standard median motor NCS were performed with surface recording from the Abductor Pollicis Brevis (APB), stimulating at the wrist and elbow. Ulnar motor NCS included surface recording from both the Abductor Digiti Minimi (ADM) and First Dorsal Interosseus (FDI) muscles, with stimulation at the wrist, below-elbow and above-elbow [7]. Standard sensory NCS included median/digit 2 and 4 and ulnar/digit 4 and 5 measurements. Trans-carpal median and ulnar orthodromic mixed nerve latencies were also measured. We expressed as a percentage the frequency of any paresthesia in the median territory on either side at Visits 1 and 2.

### Statistical analysis

The Wilcoxian signed rank test was used as the majority of the groups tested failed the Shapiro test for normality with regards to nerve conduction values of either the fractured or control side within a visit. For the sensory nerve conduction studies, we compared both median (digit 2 and 4) and ulnar (digit 4 and 5) distal sensory latencies and amplitudes between the fractured and non-fractured sides at Visits 1 and 2. We compared the median/ulnar transcarpal mixed latencies side to side at Visits 1 and 2. For the motor nerve conduction studies, we compared distal motor latency between fractured and non-fractured sides at Visit 1 and Visit 2. We performed a regression analysis to determine if there was a correlation between median nerve conduction study values and variables such as: the presence of fracture on the tested side, whether the dominant hand was fractured and if symptoms were recorded on the tested arm.

## Results

A total of 39 eligible participants with a mean age of 57.7 years (range 17–87 years) completed the first assessment between December 2016 and May 2019. The mean duration of immobilization in a cast was 6.1 weeks. Thirty (77%) of the initial participants returned for repeat assessment. The 9 patients missing at Visit 2 either found the first study too uncomfortable or were lost to follow-up. The interval between Visits 1 and 2 was 7.8 weeks. The fracture affected the dominant arm in 43.6% of participants with 92.3% being right hand dominant.

The frequency of reported paresthesia in the median territory on the fractured side was 20% at Visit 1 and 26% at Visit 2. The same frequency values for the unfractured hand were 8% at Visit 1 and 1.8% at Visit 2.

At Visit 1, there were 10 nerve conduction studies (12.8% of 78 hands tested) meeting criteria for mild median neuropathy: 4 on the fractured side, 6 on the non-fractured side. At visit 2, there were 9 studies meeting criteria for mild median neuropathy (15% of 60 hands tested): 6 on the fractured side and 3 on the non-fractured side.

Only 2 patients for the entire cohort met criteria for moderate or severe median neuropathy. Both were asymptomatic and the median nerve was affected on the non-fractured side. Motor conduction studies did not show any evidence of median neuropathy at the level of the forearm.

Table 1 shows relevant median and ulnar distal motor and sensory latency and amplitude side-to-side comparisons at Visit 1 and Visit 2. The only comparison that showed a statistically significant difference was a 0.31 ms relative prolongation of median distal motor latency on the fractured side at visit 2 but not at visit 1. This did not however achieve diagnostic significance given that ipsilateral distal median sensory latencies were not significantly prolonged on the fractured side, and these are much more sensitive indicators of median neuropathy at the wrist[8]. The regression analysis did not show a statistically significant association of nerve conduction study trends consistent with median nerve impairment and any of the 3 potential factors studied: involvement of the dominant hand, presence of sensory symptoms, or presence of ipsilateral fracture. Of these 3 factors, the presence of sensory symptoms showed a slight trend towards neuropathic abnormalities, but without reaching statistical significance.

**Table 1:** Median and ulnar motor and sensory nerve conduction studies, comparing the fractured and non-fractured limbs.

	**VISIT 1**		
	Fractured Side	Non-fractured Side	p-Value
**Median DML**	3.37	3.41	0.337
**Ulnar DML**	2.74	2.78	0.04
**Median D2 DSL**	2.63	2.67	0.71
**Median D4 DSL**	2.70	2.82	0.01
**Ulnar D4 DSL**	2.59	2.67	0.02
**Transcarpal difference**	0.18	0.16	0.91
**Median D2 amp**	37.9	39.6	0.5
**Median D4 amp**	20.8	22.1	0.25
	**VISIT 2**		
	Fractured Side	Non-fractured Side	p-Value
**Median DML**	3.54	3.23	0.0001
**Ulnar DML**	2.76	2.99	0.78
**Median D2 DSL**	2.78	2.72	0.02
**Median D4 DSL**	2.78	2.72	0.18
**Ulnar D4 DSL**	2.62	2.60	0.55
**Transcarpal difference**	0.17	0.12	0.28
**Median D2 amp**	39.6	43.0	0.08
**Median D4 amp**	20.9	21.3	0.25

DML: distal motor latency, DSL: distal sensory latency, D2,4: Digit 2,4. All latency values are expressed in milliseconds. Antidromic sensory amplitudes are expressed in microvolts.

## Discussion

The most important finding of our study is that prospective bilateral nerve conduction studies did not show a significant side-to-side difference in electrophysiologic parameters expected to correlate with focal demyelination or axonal loss in the median nerve territory on the fractured side in a cohort of 39 patients. This suggests that median nerve injury at the level of the carpal tunnel is not expected in most patients. One can postulate that acute severe symptomatic CTS in the setting of distal radius fracture may likely reflect an unusually severe acute transient angulation and compression, or an unusual degree of subsequent tissue edema with elevated compartment pressure. Another possible explanation is that such cases represent exacerbation of an underlying previously subclinical chronic entrapment.

In the largest series of 565 patients with Colle’s fracture from the Mayo Clinic, compressive neuropathy was noted in 7.9% of patients and was reported to be the most frequent complication, ahead of arthrosis, malposition-malunion, tendon rupture or shoulder-hand syndrome[9]. There was however no documentation of whether the diagnosis of CTS was supported by objective data other than the clinical interpretation of hand paresthesia or physical examination findings. There was also no mention of the baseline incidental occurrence of compressive neuropathy on the contralateral side.

In a series of 61 patients with distal radius fracture, Stark reported that electrodiagnostic studies were “abnormal” in 52.5% of fractured hands, with 15/32 of this subgroup having documented paresthesia, dysesthesia, or sensory impairment on testing[10]. A valid ring-finger antidromic median/ulnar sensory latency comparison was used, but with an unusually sensitive cut-off value of 0.3 ms, which could be expected to lead many false positives. Additionally, no comparison was made to the unfractured limb. In a population study of subjects with or without wrist fracture, Atroshi found that 18% of asymptomatic normal subjects showed electrodiagnostic evidence of median neuropathy at the wrist[11]. A more recent study of 40 hands without symptoms of CTS (CTS-6 score of 0) showed laboratory evidence of median neuropathy at the wrist at a frequency of 43% for nerve conduction studies and 23% for ultrasound[12]. The interpretation of median nerve electrodiagnostic results must thus take into account the sensitivity of distal motor and sensory latency limits chosen for the interpretation and the fact that background subclinical entrapment occurs in a substantial percentage of the general population.

Our study also revealed that paresthesia in the median territory were commonly reported by patients on the fractured side at the time of cast removal (20%) and at the routine 7- week subsequent follow-up visit (26%). This was however not associated with electrodiagnostic evidence of median nerve focal demyelination or axonal loss. A more proximal (forearm level) transient median nerve compression could also be considered in these patients, but this possibility was not supported by any abnormality on median nerve stimulation at the elbow. Only two cases could be classified as “moderate to severe” median neuropathy at the wrist in the commonly accepted electrophysiologic classification. In both cases the median nerve was abnormal on the side *contralateral* to the distal radius fracture. The hand paresthesia described by our patients could represent minor sensory axon dysfunction not detected by electrophysiologic testing, or they could be a non-specific neuropathic misinterpretation in the context of significant recent bony and soft tissue injury and immobilization in a cast. Clinicians recognize that non-localizing sensory symptoms are commonly reported by patients with tenosynovitis, joint inflammation or soft tissue injury, in the absence of objective signs of focal peripheral nerve injury.

There were limitations to our analysis. The sample size was small and may not have represented fully the wide range of velocity and angle of displacement that may occur in distal radius trauma. Although our study size may be adequate to compare side-to-side average median nerve latencies and amplitudes, it may not have the power to detect the occurrence of median neuropathy meeting electrodiagnostic criteria for CTS, which has a low expected frequency. There was a 23% drop out rate between the two assessments. The electrophysiologic technologists were not blinded to the side of fracture, though they applied rigorous institutional nerve conduction study protocols bilaterally. Our Laboratory does not routinely use the Combined Sensory Index (CSI)[13], which is a highly sensitive summative score for the detection of carpal tunnel. Though we did not include the radial-median distal sensory latency comparison (recording from the thumb), we did measure the other two elements of the CSI. No attempt was made to clinically assess median motor function. This would have been difficult to quantify, as manual muscle testing opponens pollicis or abductor pollicis brevis is often unreliable in healthy volunteers, and fraught with confounding factors in the setting of recent trauma and immobilization in a cast.

In summary, our prospective electrodiagnostic study suggests that significant median nerve injury is not expected in the course of uncomplicated distal forearm fracture. This must be contrasted with the frequent occurrence subjective symptoms of paresthesia in the median territory, which could often represent a benign non-specific manifestation of recent trauma. Electrodiagnostic studies may thus be of value in determining whether hand paresthesia truly reflect median nerve compression.
