# Correction: Systematic prospective electrophysiological studies of the median nerve after simple distal radius fracture

**DOI:** 10.1371/journal.pone.0243490

**Published:** 2020-12-03

**Authors:** Pierre R. Bourque, John Brooks, Theo Mobach, Braden Gammon, Steven Papp, Jodi Warman-Chardon

The fourth author's first name is spelled incorrectly. The correct name is: Braden Gammon.
